# Tricuspid valve disease and cardiac implantable electronic devices

**DOI:** 10.1093/eurheartj/ehad783

**Published:** 2023-12-14

**Authors:** Martin Andreas, Haran Burri, Fabien Praz, Osama Soliman, Luigi Badano, Manuel Barreiro, João L Cavalcante, Tom de Potter, Torsten Doenst, Kai Friedrichs, Jörg Hausleiter, Nicole Karam, Susheel Kodali, Azeem Latib, Eloi Marijon, Suneet Mittal, Georg Nickenig, Aldo Rinaldi, Piotr Nikodem Rudzinski, Marco Russo, Christoph Starck, Ralph Stephan von Bardeleben, Nina Wunderlich, José Luis Zamorano, Rebecca T Hahn, Francesco Maisano, Christophe Leclercq

**Affiliations:** Department of Cardiac Surgery, Medical University of Vienna, Level 7C, Waehringer Guertel 18-20, Vienna 1090, Austria; Cardiac Pacing Unit, Cardiology Departement, University Hospital of Geneva, Geneva, Switzerland; Bern University Hospital, University of Bern, Bern, Switzerland; Discipline of Cardiology, SAOLTA Healthcare Group, Galway University Hospital, Health Service Executive, and University of Galway, Galway H91 YR71, Ireland; Department of Medicine and Surgery, University of Milano Bicocca, Milan, Italy; Department of Cardiology, Istituto Auxologico Italiano, IRCCS, Milan, Italy; Cardiology Department, Hospital Universitario Alvaro Cunqueiro, Instituto de Investigación Sanitaria Galicia Sur (IISGS), Vigo, Spain; Cardiac MR and Structural CT lab, Allina Health Minneapolis Heart Institute at Abbott Northwestern Hospital, Minneapolis, MN, USA; Cardiovascular Center, OLV Hospital, Aalst, Belgium; Department of Cardiothoracic Surgery, Friedrich-Schiller-University of Jena, Jena University Hospital, Jena, Germany; Clinic for General and Interventional Cardiology/Angiology, Heart and Diabetes Center North Rine Westphalia, Bad Oeynhausen, Germany; Medizinische Klinik I, Ludwig-Maximilians-University, Munich, Germany; German Center for Cardiovascular Research (DZHK), Partner Site Munich Heart Alliance, Munich, Germany; Cardiology Department, European Hospital Georges Pompidou, Université Paris Cité, Paris, France; Division of Cardiology, Department of Medicine, New York-Presbyterian/Columbia University Irving Medical Center, NewYork, NY, USA; Montefiore Einstein Center for Heart and Vascular Care, Montefiore Medical Center, NewYork, NY, USA; Cardiology Department, European Georges Pompidou Hospital, Paris, France; Department of Cardiology, The Valley Health System, the Synder Comprehensive Center for Atrial Fibrillation, Ridgewood, NJ, USA; Herzzentrum Medizinische Klinik und Poliklinik II, Universitätsklinikum Bonn, Bonn, Germany; Department of Cardiology, Guy’s & St Thomas’ NHS Trust, London, UK; Department of Coronary and Structural Heart Diseases, National Institute of Cardiology in Warsaw, Warsaw, Poland; Department of Cardiac Surgery and Heart Transplantation, Azienda Ospedaliera San Camillo Forlanini, Rome, Italy; Department of Cardiothoracic and Vascular Surgery, German Heart Center of Charité, Berlin, Germany; Department of Cardiology, Universitätsmedizin Mainz of the Johannes Gutenberg-University of Mainz, Mainz, Germany; Department of Cardiology/Angiology, Asklepios Klinik Langen, Langen, Germany; Department of Cardiology, University Hospital Ramon y Cajal, Madrid, Spain; Division of Cardiology, Department of Medicine, New York-Presbyterian/Columbia University Irving Medical Center, NewYork, NY, USA; Heart Valve Center, Cardio-Thoracic-Vascular Department, IRCCS San Raffaele Scientific Institute, Milan, Italy; Department of Cardiology, University of Rennes, CHU Rennes, lTSI-UMR1099, Rennes F-35000, France

**Keywords:** Pacemaker, Cardiac implantable electronic device, Tricuspid regurgitation, Lead related

## Abstract

The role of cardiac implantable electronic device (CIED)-related tricuspid regurgitation (TR) is increasingly recognized as an independent clinical entity. Hence, interventional TR treatment options continuously evolve, surgical risk assessment and peri-operative care improve the management of CIED-related TR, and the role of lead extraction is of high interest. Furthermore, novel surgical and interventional tricuspid valve treatment options are increasingly applied to patients suffering from TR associated with or related to CIEDs. This multidisciplinary review article developed with electrophysiologists, interventional cardiologists, imaging specialists, and cardiac surgeons aims to give an overview of the mechanisms of disease, diagnostics, and proposes treatment algorithms of patients suffering from TR associated with CIED lead(s) or leadless pacemakers.

## Graphical Abstract

**Graphical Abstract:**
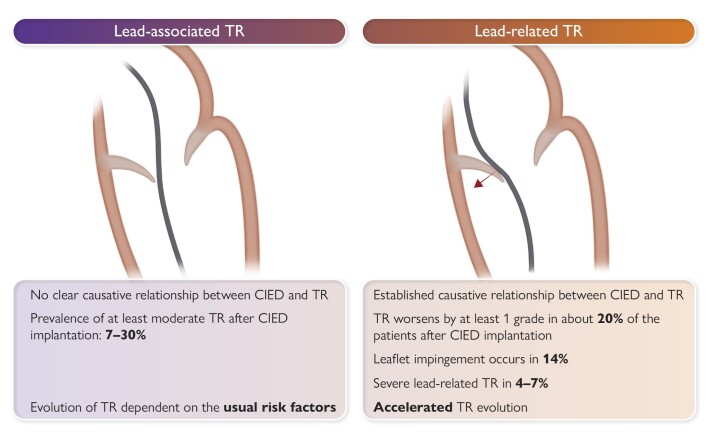
Definition of TR in the presence of a CIED lead. CIED, cardiac implantable electronic device; TR, tricuspid regurgitation.

## Introduction

The implantation of cardiac implantable electronic devices (CIEDs) has increased exponentially. More than 3.8 permanent pacemakers (PPMs) per million inhabitants, 2.2 implantable cardioverter defibrillators (ICD), and 1.8 cardiac resynchronization therapy (CRT) devices are implanted yearly in Europe.^1^ Tricuspid regurgitation (TR) associated with CIEDs and worsening TR after PPM are increasingly recognized as relevant clinical conditions linked to a higher risk of heart failure and mortality.^2,3^ Interactions between the tricuspid valve (TV) and CIED lead(s) include mechanical interference or damage to the apparatus,^4,5^ as well as pacing-related ventricular remodelling.^6–8^ Patients with worsening TR after PPM have an increased long-term mortality.^3^ Novel surgical and interventional TV treatment options are emerging and may be applied to patients suffering from TR related to or associated with CIEDs. This multidisciplinary review article was developed with electrophysiologists, interventional cardiologists, imaging specialists, and cardiac surgeons during a structured discussion process to provide clinical guidance (see Supplementary data online, *Section S1*).

## Definition and terminology

**Figure 1:**
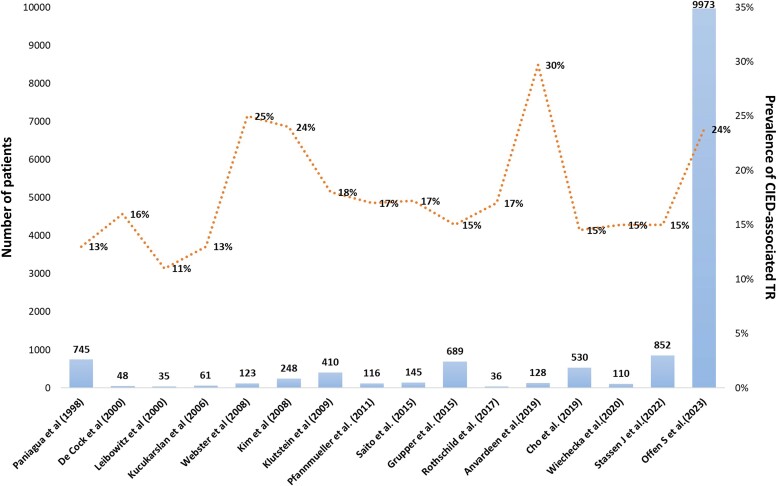
Prevalence of cardiac implantable electronic device-associated tricuspid regurgitation in several studies. CIED, cardiac implantable electronic device; TR, tricuspid regurgitation.

Transvenous ICDs, CRT devices, and PPMs are typically implanted with a lead that crosses the TV and anchored in the right ventricle, while leadless cardiac pacemakers (LCPMs) are directly placed into the right ventricle. Multiple reports have described interferences between CIED leads or LCPM and the TV apparatus (*Table 1* and *Figure 1*), pacing itself resulting in TR, or more rarely provoking tricuspid stenosis.^24^ While in many patients, CIED and TR coexist (CIED-associated TR), a causal relationship should be assumed when TR of any grade appears or pre-existing TR worsens following right ventricular (RV) lead insertion.^25^ Further, demonstration of well-delineated lead–leaflet interaction also suffices to diagnose CIED-related TR (*Graphical Abstract*). For the subset of patients in whom causality can be established, we propose to use the term of CIED-related TR. Of note, at more advanced disease stages, the differentiation between lead-related and lead-associated TR may no longer be possible because of the predominance of RV remodelling.

**Table 1:** Prevalence and incidence of cardiac implantable electronic device-associated tricuspid regurgitation in several studies

Author of study	Year	Journal	No. of pts. with CIED	Country of origin	Study design	Cohort	Imaging modality	Prevalence and/or incidence of CIED-associated TR (%)
De Cock *et al*.^9^	2000	*Pacing and Clinical Electrophysiology*	96	Netherlands	Prospective	**Study group (*n* = 48):** 24 pts. − 2 ventricular leads + 1 atrial lead; 15 pts. − 2 atrial leads + 1 ventricular lead; 9 pts. − 2 two ventricular & 2 atrial leads. **Controls**-Equal number of pts matched for age, gender, indications for cardiac stimulation with DDD pacemakers, and duration of stimulation	Doppler echocardiography	Prevalence of TR: 20.8% (20/96 patients)
Leibowitz *et al*.^10^	2000	*Cardiology*	35	Israel	Prospective	35 consecutive patients referred to the electrophysiology service for the implantation of either permanent pacemakers or automatic implantable cardioverter defibrillators (ICDs)	2D and Doppler echocardiography	Incidence of worsened TR by one grade: 11.4% (4/35 patients)
Kucukarslan *et al*.^11^	2006	*Journal of Cardiac Surgery*	61	Turkey	Prospective	Patients referred for the implantation of either permanent PM or ICD	M-mode, 2D, and Doppler echocardiography	Incidence of worsened TR by one grade: 13.1% (8/61 patients)
Webster *et al*.^12^	2008	*Journal of Interventional Cardiac Electrophysiology*	123	USA	Retrospective chart review	At least one transthoracic echocardiographic study before the placement of the transvenous lead and at least one echo afterwards	Echocardiography	TR increased by one grade in 22%, two grades in 3%
Kim *et al*.^13^	2008	*Journal of the American Society of Echocardiography*	248	USA	Retrospective chart review	Patients with pre- and post-implantation echocardiograms	Echocardiography	TR worsened by one grade or more: 24%, Severe TR developed in 3.9%
Klutstein *et al*.^14^	2009	*Pacing and Clinical Electrophysiology*	410	Israel	Retrospective chart review	Patients undergoing (permanent pacemaker implantation) PPI who had Doppler echocardiograms performed before and after PPI	Doppler echocardiography	TR worsened by ≥2 grades: 18.3% (75/410 patients)
Pfannmueller *et al*.^15^	2011	*European Journal of Cardio-thoracic Surgery*	116	Germany	Retrospective chart review	Patients with a previously implanted permanent ventricular pacemaker lead (PPL) and significant TR who underwent tricuspid valve (TV) surgery. Indications for isolated TV surgery were symptomatic severe tricuspid regurgitation (TR) while indications for TV surgery were symptomatic severe TR alone or at least mild-to-moderate TR with marked tricuspidal annular dilation in echocardiography (>4.0 cm) in patients with other indications for cardiac surgery.	Echocardiography	Prevalence of lead-related TR: 7% (8/116 patients).
Saito *et al*.^6^	2015	*The American Journal of Cardiology*	145	Australia	Prospective	Patients with persistent high grade atrioventricular block (defined as 2:1 atrioventricular block or higher) and sinus rhythm, scheduled to undergo dual-chamber pacemaker implantation	Transthoracic echocardiography	TR increased by one grade in 17.2%, two grades in 4.8%
Al-Bawardy *et al*.^16^	2015	*Pacing and Clinical Electrophysiology*	1596	USA	Prospective	Patients with first-time devices implantation which included ICD or PPM. One pre-implantation echocardiogram and at least one post-implantation echocardiogram.	Echocardiography	Prevalence of severe TR pre-implantation was 27%
Grupper *et al*.^17^	2015	*The American Journal of Cardiology*	689	Israel and USA	Retrospective analysis	Patients who underwent implantation of cardiac resynchronization therapy (CRT) or upgrade of other devices to CRT	Echocardiography	TR increased by at least one grade in 15%
Rothschild *et al*.^18^	2017	*Journal of Interventional Cardiac Electrophysiology*	36	USA	Prospective	Patients referred for PPM implantation	Limited 2D and colour Doppler transthoracic echocardiography (TTE)	TR increased by at least one grade in 17%
Anvardeen *et al*.^19^	2019	*CJC Open*	128	Canada, Australia, China	Prospective	Patients with pre-procedural echocardiograms within 48 h before lead implantation and follow-up echocardiograms at 4–6 weeks, 6 months, and 1 year after the procedure.	Echocardiograms (both 2D and 3D)	New or worsened TR: 29.7% (38/128), TR increased by only 1 grade: 25% (32/38)
Cho *et al*.^20^	2019	*Pacing and Clinical Electrophysiology*	530	South Korea	Retrospective study	Patients admitted for PM implantation with baseline echocardiography and then 3 months post index procedure in OPD and yearly thereafter.	2D and Doppler echocardiography	Incidence of moderate to severe TR: 14.5%; of which isolated moderate to severe TR: 48.1%
Wiechecka *et al*.^21^	2020	*Cardiology Journal*	110	Poland	Observational, retrospective study,	Patients after first CIED implantation (PPM, ICD, or CRT), who had echocardiographic assessment of TR and PE before and after the procedure. Only patients with echocardiogram performed <60 days before and up to 7 days after implantation were included	2D transthoracic echocardiography	Acute new or worsened TR: 15.5%
Stassen J *et al*.^22^	2022	*Europace*	852	Netherlands, Belgium, Finland,	Retrospective study	Patients with baseline and at least 6 months echocardiogram after CRT	Transthoracic echocardiography	TR worsened in 85 patients (9.9%)
Offen S *et al*.^23^	2023	*International Journal of Cardiology*	9973	Australia	Retrospective study	Patients from a large multicentric echocardiographic registry (25 Australian centers)	Echocardiography	5490 (29.2%) mild TR; 3068(16.3%) moderate TR; and 1415 (7.5%) severe TR

CIED, cardiac implantable electronic device; CRT, cardiac resynchronization therapy; ICD, implantable cardioverter defibrillator; PPI, permanent pacemaker implantation; PPM, permanent pacemaker; TR, tricuspid regurgitation; TTE, transthoracic echocardiography; TV, tricuspid valve.

### Mechanisms

**Figure 2:**
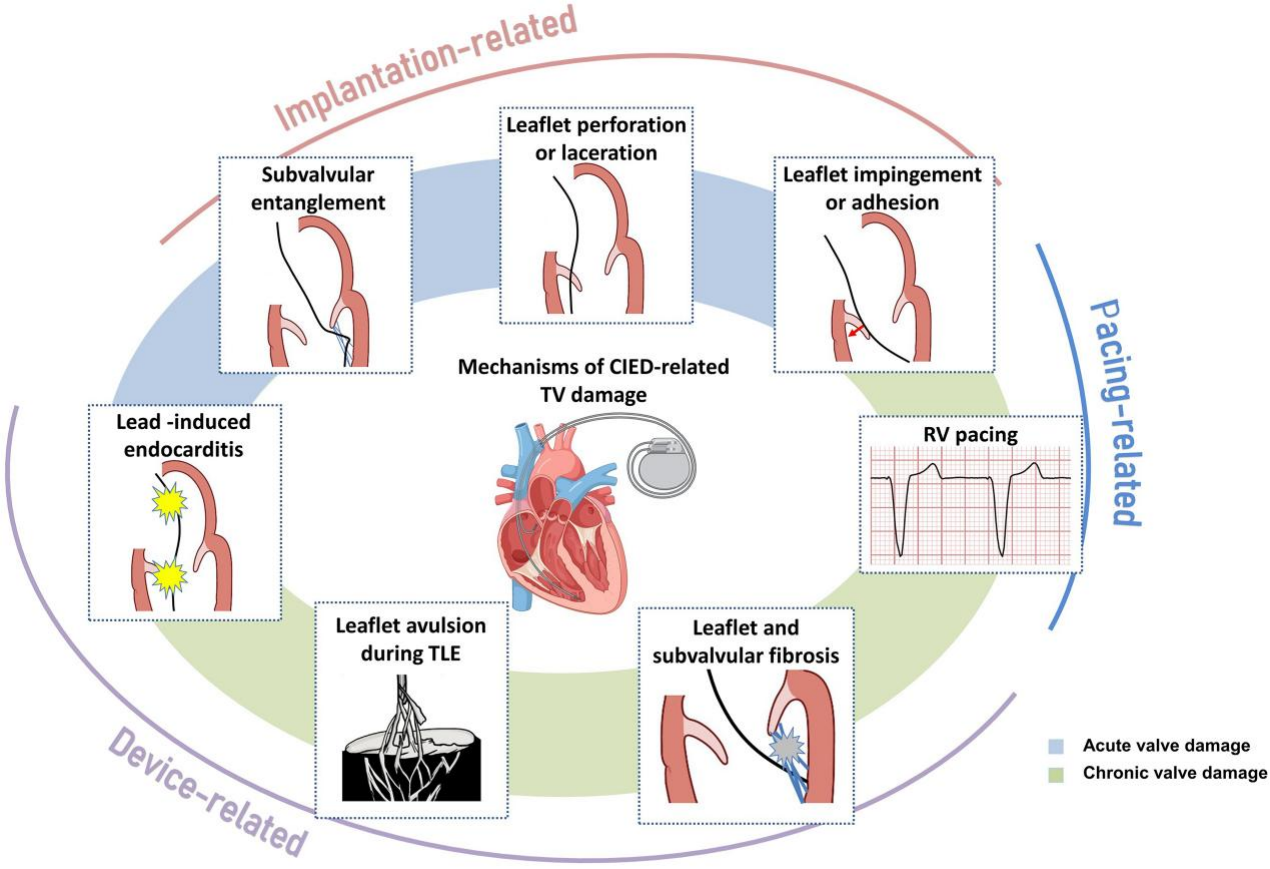
Mechanisms of cardiac implantable electronic device-related tricuspid regurgitation. CIED, cardiac implantable electronic device; RV, right ventricle; TLE, transvenous lead extraction; TV, tricuspid valve.

**Figure 3:**
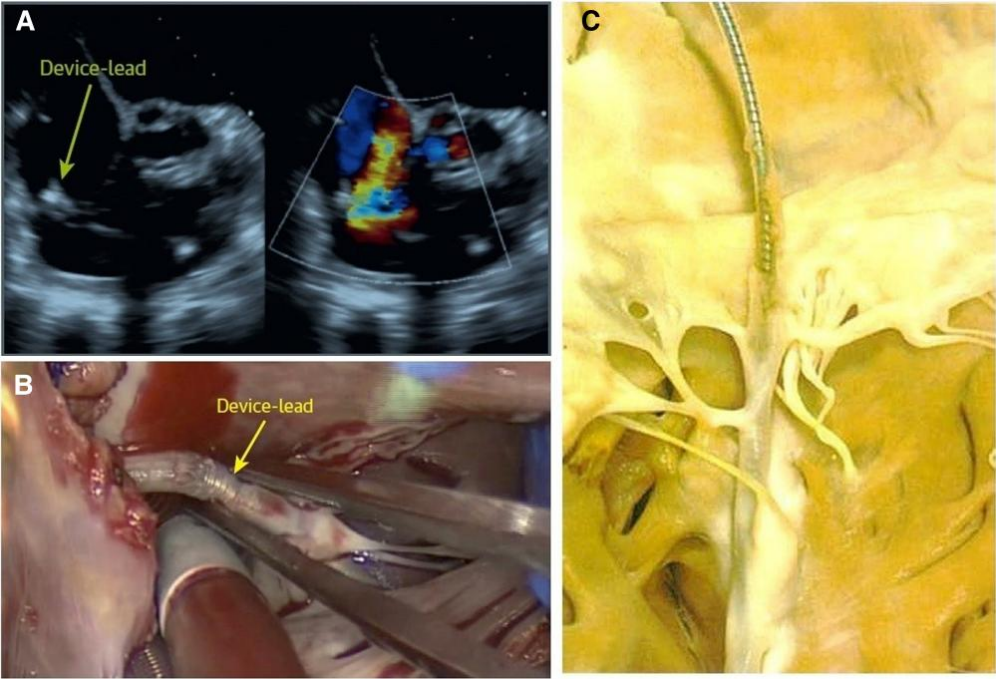
Surgical situ in a patient with lead-related tricuspid regurgitation. (*A*) Device lead attached to the tricuspid valve in echocardiography; (*B*) intra-operative view of an attached cardiac implantable electronic device lead; and (*C*) device lead attached to the tricuspid valve and the subvalvular apparatus with significant ingrowth.

Although previously categorized as a ‘primary’ cause of TR, the presence of a lead across the TV and the multiple mechanisms of TR in the presence of a CIED have led investigators to reclassify CIED-related TR as a separate aetiologic entity, since this phenotype necessitates specific work-up and dedicated multidisciplinary management.^26,27^ The mechanisms responsible for CIED-related TR can be divided into three categories: implantation related, pacing related, and device related (*Figure 2*). In vivo 2D and 3D echocardiography and post-mortem examinations have revealed that leads can interfere with the TV apparatus by impinging on a leaflet or adhering to it, interfering with the subvalvular apparatus or perforating/lacerating a leaflet (*Figure 3A* and *B* and Supplementary data online, *Video S1*).^2,28^ In addition, following transvenous lead extraction (TLE), TR can be the consequence of leaflet avulsion or chordal rupture. Acute leaflet impingement has been observed in 14% of the patients after lead placement, mainly affecting the septal leaflet.^18^ In addition, the presence of a CIED lead might predispose to thrombus formation or endocarditis.^4^

**Figure 4:**
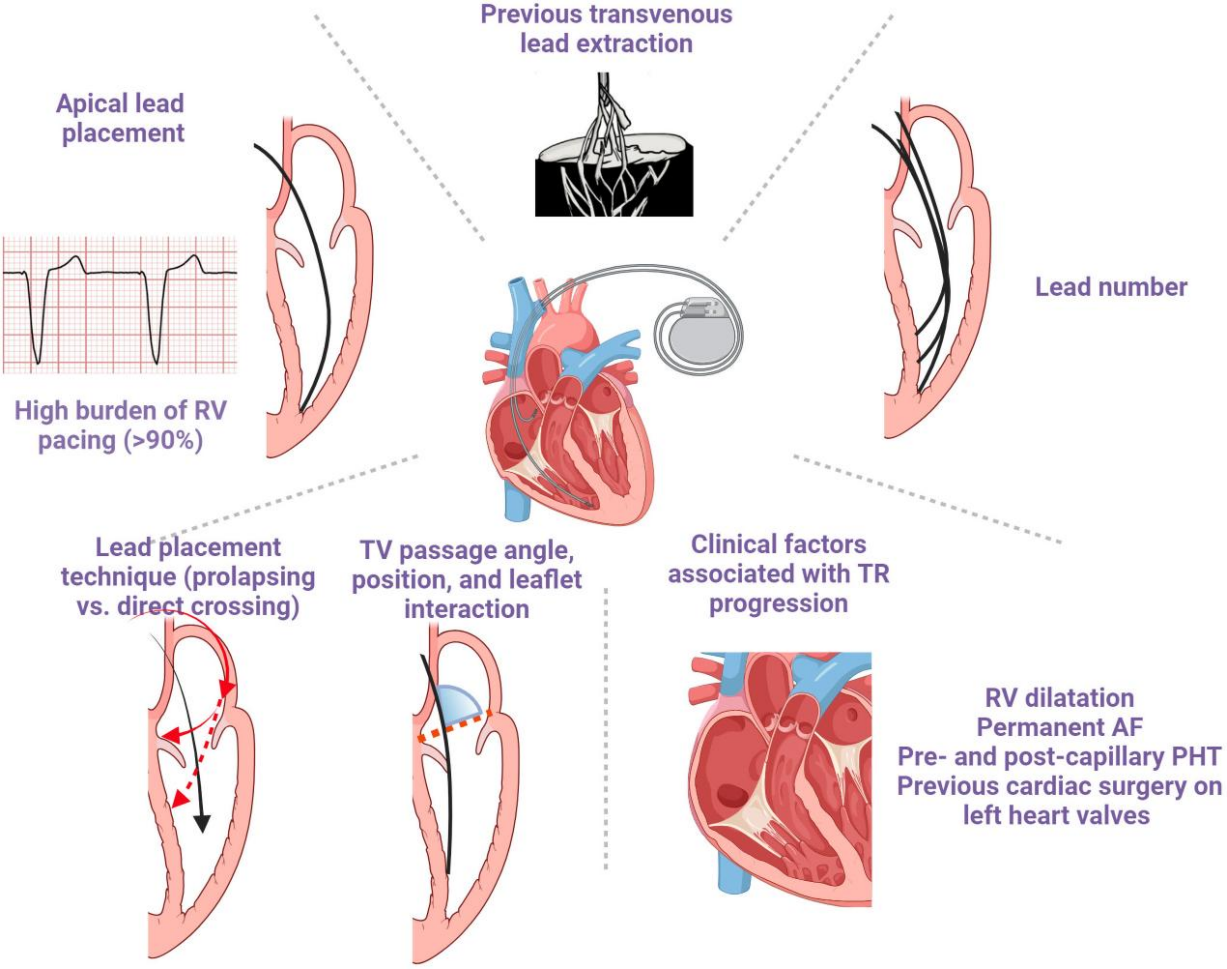
Risk factors of cardiac implantable electronic device-related tricuspid regurgitation. AF, atrial fibrillation; PHT, pulmonary hypertension; RV, right ventricle; TV, tricuspid valve.

Other risk factors include TV lead passage angle and increasing number of leads (*Figure 4*).^9,29^ While observational studies have suggested that apical lead placement is more likely to impinge the posterior leaflet,^4,29^ a randomized study allocating patients 1:1:1 to RV apex, RV septum, and coronary sinus lead implantation failed to confirm this finding. Notwithstanding, 3D echocardiography showed that commissural or central lead placement prevents leaflet impingement.^30,31^ Comparison with RV ICD implants did not support an impact of lead type.^19,32^

The timing of TR progression following CIED implantation depends on its mechanism. Acute TR changes are likely the result of mechanical leaflet impingement/restriction or injury to the TV apparatus. Exacerbation of TR following CIED placement may be detected between 1 and 12 months,^5,33^ while heart failure hospitalization generally occurs beyond 12 months.^34^ However, substantial cardiac inflammatory alterations have also been observed within days after the procedure (*Figure 3C*).^4,35–37^

Importantly, all other clinical conditions contributing to TR progression including permanent atrial fibrillation (AF), pre- and post-capillary pulmonary hypertension, RV dilatation, and previous cardiac surgery on left heart valves equally play a role and require specific attention (*Figure 4*).

#### The role of the pacing strategy

The pacing strategy has not been consistently demonstrated to affect TR, but recently developed technologies might change clinical perception. New-onset TR has been rarely reported following His-bundle pacing, while TR reduction has been more frequently documented.^38^ This may be explained by lead implantation into the atrial aspect of the tricuspid annulus or in the antero-septal commissure.^38,39^ With left bundle branch area pacing, lead placement >16–19 mm away from the tricuspid annulus was associated with less TR.^40–42^ In another series, an overall decrease of TR was seen at 1 year (11% worsened and 31% improved).^43^ Conduction system pacing may also prevent TR by avoiding dyssynchronous contraction^38^ and minimizing interaction through the use of thinner and lighter pacing leads.

Leadless cardiac pacemaker implantation does not preclude TR development, and acute procedure-related TV damage may occur. However, damage of the subvalvular apparatus by the fixation tines was unlikely and the number of required deployments did not predict TR.^5,44,45^ An observational 12-month follow-up study of 53 patients showed an increase in TR in 23 patients (43%) without difference compared with dual chamber PPM (38%). Notwithstanding, a more septal position of the device and implantation close to the TV was associated with TR worsening.^5,45^ Tricuspid regurgitation has also been linked to single-chamber RV pacing,^46–49^ presumably due to the alteration of the RV geometry.^50^

### Prevalence

In 1974, an autopsy first revealed lead-related TR due to the perforation of the anterior TV leaflet.^51^ Due to the heterogenicity of methodology and population, studies have reported extremely variable prevalence of lead-associated TR (7% to 30%; *Figure 1* and *Table 1*).^6,9–23,46,47,52–55^ In a recent large-scale multicentre prospective cohort study, CIED-related TR accounted for 5% of all severe TR cases.^56^ Tatum *et al*.^57^ reported that lead placement is associated with post-procedural TR worsening in 20% of the patients with a total prevalence of at least moderate TR in 41%.

However, studies are limited by retrospective design, small sample sizes, and variable follow-up. Importantly, different echocardiographic techniques (CIED-related TR is more difficult to diagnose using 2D than 3D echocardiography^30^), various definitions of ‘significant’ post-procedural TR, and inconsistent TR grading methods have been used.^4^ In several of these reports, no systematic assessment of TR mechanism has been performed and pre-procedural echocardiography was missing.

### Natural history and outcome

**Figure 5:**
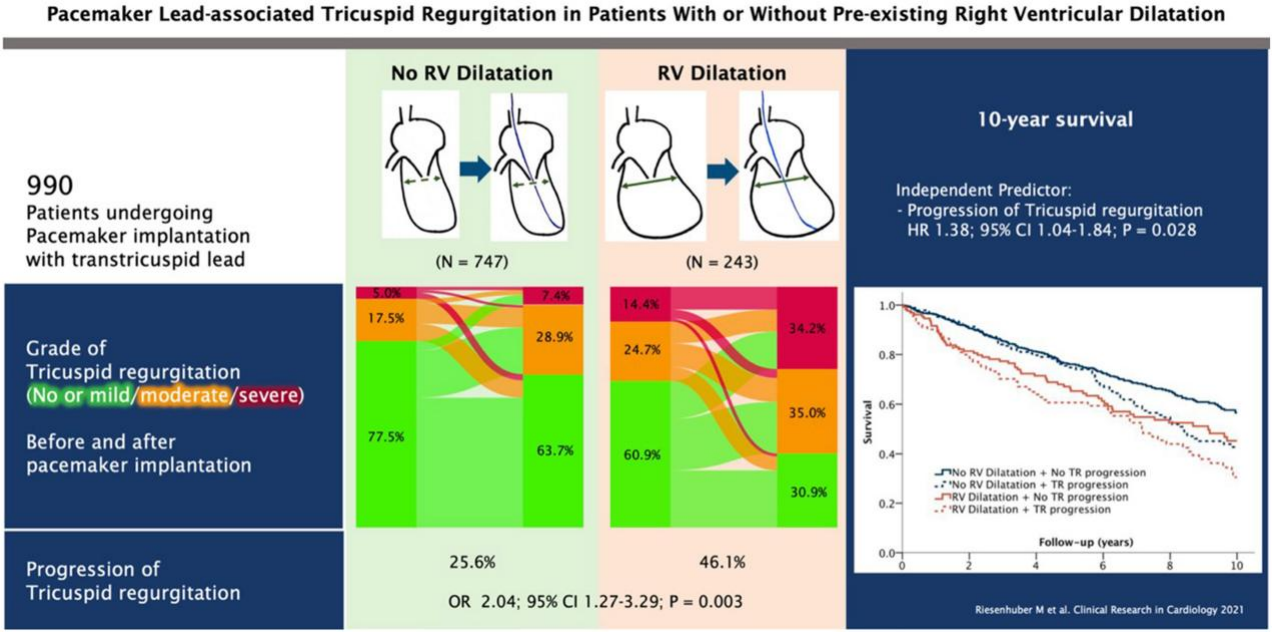
Risk of lead-associated tricuspid regurgitation after pacemaker implantation. CI, confidence interval; HR, hazard ratio; *n* = number; OR, odds ratio; RV, right ventricle; TR, tricuspid regurgitation (open license for re-print).^3^

The natural course and prognosis of CIED-related TR do not differ from other TR phenotypes^58^ and result in right heart remodelling, with increased right atrial and RV volumes, impaired RV function, and potentially increased mortality.^3,52,59^ Symptoms and signs are those associated with severe TR such as fatigue, dyspnoea, hepatomegaly, ascites, and peripheral oedema.^60^ CIED-related severe TR has been linked to heart failure hospitalization, TV surgery, or CRT upgrading, as well as poorer long-term survival.^34,52^ Stassen *et al*.^22^ suggested that improvement of CIED-related TR during follow-up was associated with better outcome. In analogy to secondary TR, a step-wise increase in the adjusted risk of mortality according to TR severity was reported in 18 800 patients with a CIED lead.^23^ Moderate or severe TR was more prevalent (23.8% vs. 7.7%) in individuals with CIEDs compared with those without and was linked to a 1.6- to 2.5-fold increase in all-cause mortality after adjustment for age, sex, AF, or left-heart disease. Riesenhuber *et al*.^3^ reported an increased risk of TR development in patients with a dilated RV undergoing PPM implantation and confirmed reduced survival in patients with TR progression (*Figure 5*).

### Diagnosis of cardiac implantable electronic device-related tricuspid regurgitation

#### Echocardiography

**Figure 6:**
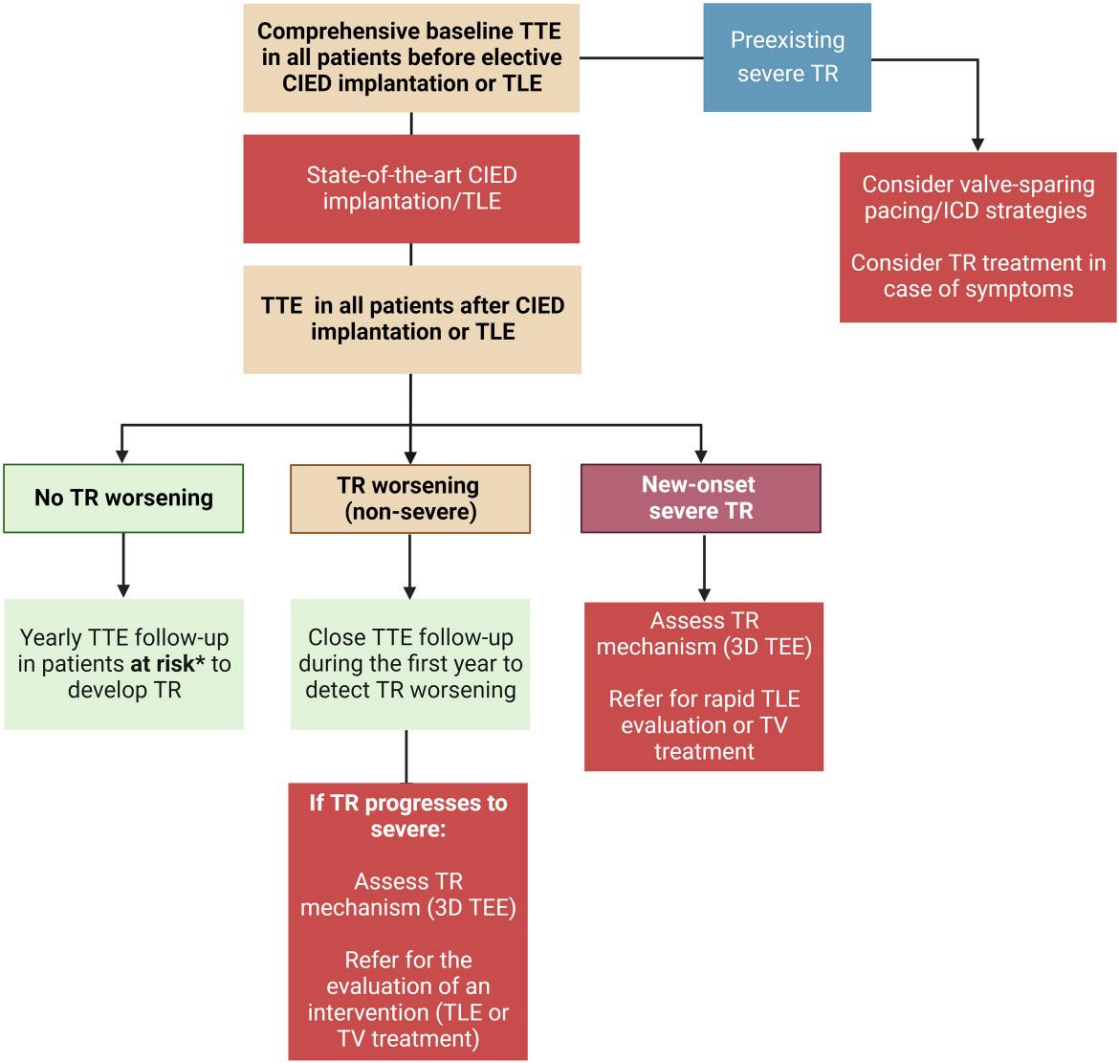
Imaging algorithm for patients undergoing cardiac implantable electronic device implantation or transvenous lead extraction. CIED, cardiac implantable electronic device; TEE: transoesophageal echocardiography; TLE, transvenous lead extraction; TR: tricuspid regurgitation; TTE, transthoracic echocardiography; TV, tricuspid valve.

Timely recognition of new or worsening TR after device implantation can be challenging if baseline echocardiography before implantation is not available. Ideally, candidates for CIED implantation should have a comprehensive baseline echocardiography before CIED implantation with a focus on TV and RV function (*Figure 6*). This step is of particular importance in patients cumulating risk factors for TR progression. In case of severe TR at baseline, an interdisciplinary discussion with valve specialists should be scheduled to consider valve-sparing pacing or ICD strategies facilitating future TR treatment.

A complete echocardiographic study should be obtained within the first weeks after CIED implantation. In the presence of notable TR worsening, transoesophageal echocardiography (TEE) is the imaging modality of choice to assess CIED-related TR and differentiate it from incidental CIED-associated TR (*Table 2*).^4,61^ Given near-field imaging and advances of 3D imaging resolution, transthoracic echocardiography (TTE) may be a reasonable alternative to determine the location of the lead.^30,61^ Reconstruction of 3D images, either from TTE or TEE, may allow for imaging from the atrial or ventricular aspect of the device, determine the trajectory of the CIED lead, and assess leaflet and lead motion. Defining the type and extent of interaction may help anticipate the risk of TLE.^62,63^ Impingement is characterized by normal diastolic excursion of the TV leaflet with separation from the PPM lead but apposition of the leaflet and lead in systole with reduced systolic leaflet excursion. Adhesion is diagnosed when leaflet and lead move together throughout the cardiac cycle with reduced systolic leaflet excursion. Perforations may be seen on 3D en-face reconstruction of the leaflet surface.

**Table 2:** Mechanism of cardiac implantable electronic device-related tricuspid regurgitation by 3D echocardiography

Mode of TR	Echocardiographic findings	Example
Impingement	Normal diastolic excursion of the tricuspid valve leaflet with separation from the PPM lead Systolic apposition of the leaflet and lead with reduced systolic leaflet excursion.	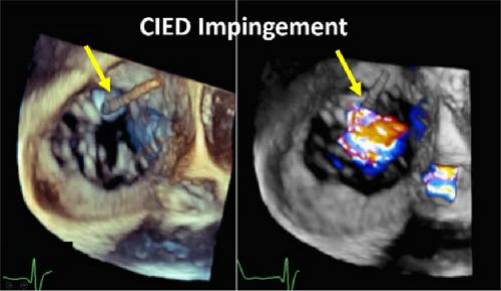
Adhesions	PPM lead adhesion is diagnosed when the leaflet and lead move together throughout the cardiac cycle. Reduced systolic leaflet excursion.	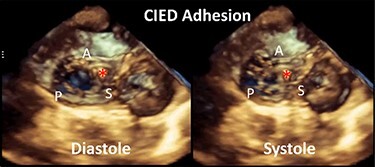
Perforation	Defect in the body of the leaflet with PPM lead traversing the defect.	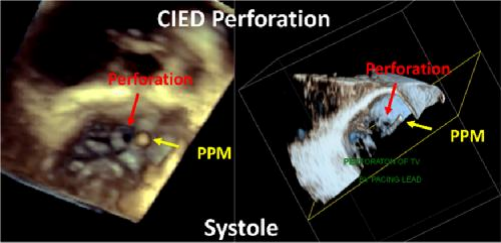
Subvalvular	Direct interference with chordae resulting in abnormal leaflet closure.	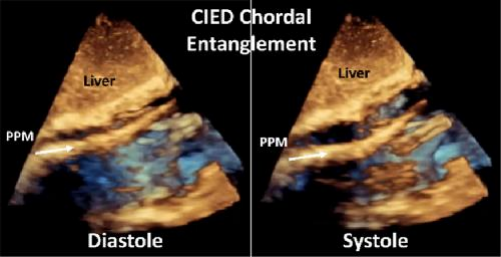

CIED, cardiac implantable electronic device; PPM, permanent pacemaker; TR, tricuspid regurgitation.

Regular annual follow-up echocardiography should be considered in patients at risk of developing TR due to coexisting clinical risk factor or RV dilatation. If moderate or severe TR is detected, referral to a Heart Valve Center with expertise in TR treatment is recommended.

#### Computed tomography

Functional cardiac computed tomography (CT) with high temporal resolution, full cardiac cycle coverage, and proper contrast protocol for right chamber enhancement may play an important complementary role to define the post-CIED TR mechanism. If TLE is considered, assessment of the vascular access routes including identification of lead fibrosis and vein stenoses may help to predict TLE complexity and anticipate potential complications.^62,64^ In addition, a detailed analysis of the tricuspid annulus and its relationship to leads and LCPM, as well as the identification of potential lead–leaflet interaction, is useful to determine the mechanism of CIED-related TR. Assessment of the extent of leaflet tethering (including height, area, tenting, and angle), position and number of leaflets, and their anatomical relations with CIED lead position improve TR intervention planning, mainly by determining the landing zone for implantable transcatheter valves and anticipating the need for lead jailing.^65^ However, CIED leads can cause significant blooming artefacts that may render the analysis of TR mechanism and lead position challenging.

#### Cardiac magnetic resonance imaging

Evaluation by cardiac magnetic resonance (CMR) for patients with TR and CIED has been shown at 1.5 T to be safe for both conditional and non-conditional (i.e. ‘legacy’) devices. However, devices must be systematically interrogated prior to and after CMR. Artefacts are related to CIED type and size and are worst in CRT-D and subcutaneous ICD.^66^ End-inspiration imaging, left arm raise, and fast gradient recall echo with short echo time for cine and wideband late gadolinium enhancement for assessment of myocardial fibrosis are techniques used to minimize artefacts. While visualization of the tricuspid leaflets is often difficult, quantification of baseline and post-intervention RV size, function, reverse remodelling, fibrosis, and eventually TR remain possible and are indicated in case of inconclusive assessment of TR severity or RV volumes and function by echocardiography.^67^

### Preventive strategy including valve sparing

#### State-of-the-art cardiac implantable electronic device/lead implantation

During lead placement, procedural variables may increase the likelihood of TV damage. As a result, the ‘prolapsing technique’ (in which the body of the wire prolapses against the leaflets and enters the RV before the lead tip) may reduce the risk of perforation and laceration compared with the ‘direct crossing technique’ (in which the tip of the lead is advanced directly across the TV towards the RV apex). Tined leads may snag on the subvalvular apparatus during placement and cause damage when being freed. Large diameter and stiff leads are likely to cause more interaction with leaflet motion than thin and flexible leads, as is the presence of the shock coil of ICD leads, although a comparison of TR between ICD and pacemaker leads is inconclusive.^57^

**Figure 7:**
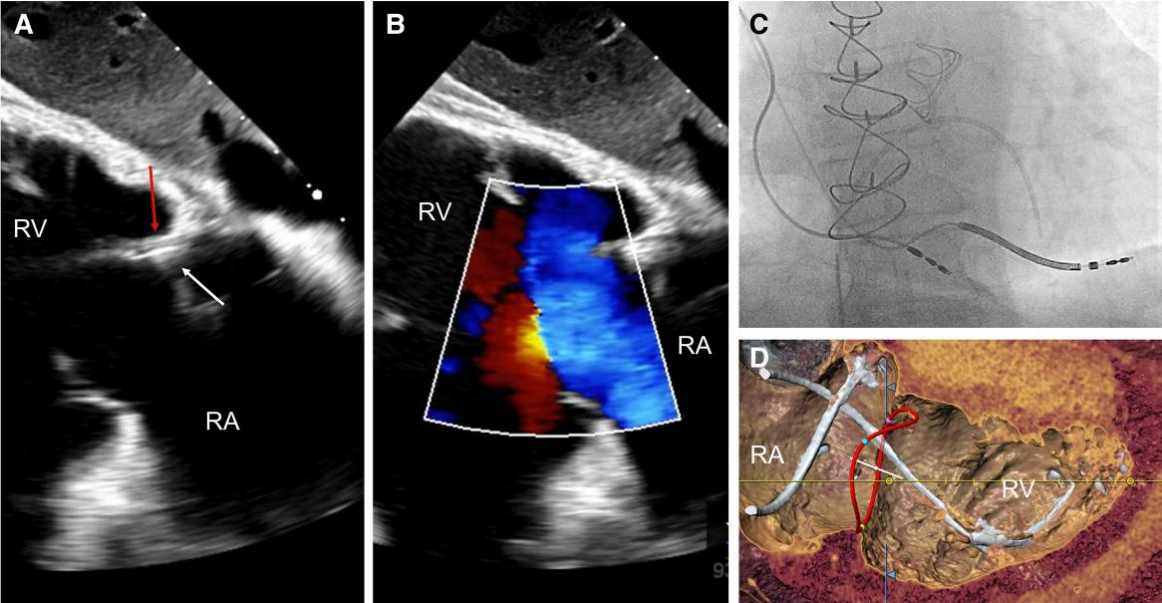
Example of a lead-related tricuspid regurgitation with ‘tricuspid kick’ and impingement of the posterior leaflet. Severe lead–leaflet interaction with impingement of the posterior leaflet resulting in severe cardiac implantable electronic device-related tricuspid regurgitation. (*A*) Impingement of the posterior leaflet; (*B*) severe tricuspid regurgitation with large coapation gap and secondary leaflet tethering; (*C*) excessive slack with visible tricuspid kick as a sign of potential interaction with the tricuspid annulus; and (*D*) cardiac computed tomography shows interaction with the tricuspid valve annulus (reconstructed line), as well as excessive slack in the right ventricle. RA, right atrium; RV, right ventricle.

Although it has been proposed to perform device implantation guided by intra-operative echocardiography, this was not shown to reduce TR in a randomized study using TTE.^68^ In a pilot observational study, TEE-guided lead implantation under deep sedation was associated with less worsening of TR at discharge, but this strategy is difficult to implement in daily practice.^69^ Fluoroscopic markers of lead impingement (e.g. tricuspid ‘kick’ on the lead body) may help to adjust lead slack and position at implantation (*Figure 7* and Supplementary data online, *Video S2*). However, these empiric markers have not been validated.^70^

#### Alternative ‘valve-sparing’ pacing/implantable cardioverter defibrillator strategies

Alternative strategies to a standard pacemaker or ICD lead placement may be considered to preserve valve function. This includes the following options (*Table 3*):

1. LCPM (see Supplementary data online, *Section S2*)
2. Surgical placement of epicardial leads
3. His-bundle pacing from the atrial aspect of the tricuspid annulus^42^
4. Left univentricular pacing via the coronary sinus^71^
5. Stimulation of the atrialized portion of the ventricle after verification of the absence of P-wave oversensing.^73^
6. Subcutaneous ICDs (see Supplementary data online, *Section S3*)
7. Alternative ICD lead placement (e.g. epicardial; see Supplementary data online, *Section S4*)

The choice of the most appropriate pacing strategy in patients with pre-existing relevant TR requires careful interdisciplinary discussion.

**Table 3:** Valve-sparing pacing and implantable cardioverter defibrillator strategies

Pacemakers	ICDs
Epicardial leads CS lead^71^ His-bundle pacing (atrial side) Leadless pacemaker*	S-ICD/EV-ICD Standalone ICD coil in the azygos vein or CS (connected to the RV port of a DF-1 ICD) coupled with anterolateral subcutaneous ‘SQ’ array + epicardial/CS pacing lead (connected to IS-1 RV port)^72^ ICD lead in middle cardiac vein (check for absence of diaphragmatic myopotential over-sensing)^73^ DF-1 ICD lead in low right atrium with epicardial or CS pacing lead^72^ Epicardial SQ-array + pacing lad or ICD lead on epicardium.

CS, coronary sinus; ICD, implantable cardioverter defibrillator; EV-ICD, extra-vascular ICD; RV, right ventricle; S-ICD, subcutaneous ICD.

### Tricuspid regurgitation treatment in patients with permanent pacemaker

**Figure 8:**
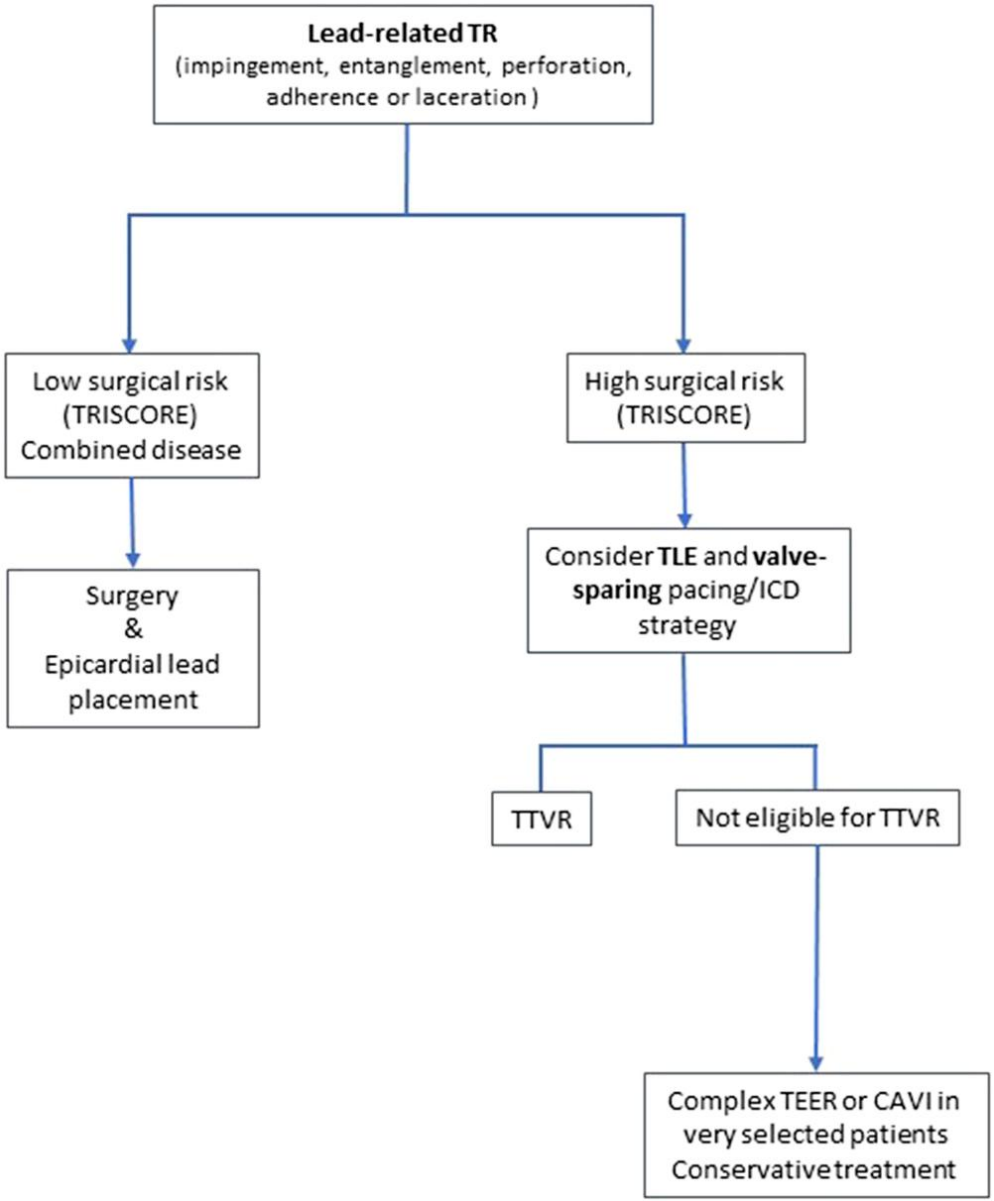
Treatment algorithm of cardiac implantable electronic device-related tricuspid regurgitation. CAVI, caval valve implantation; ICD, implantable cardioverter defibrillator; TEER, transcatheter edge-to-edge repair; TLE, transvenous lead extraction; TR, tricuspid regurgitation; TTVR, transcatheter tricuspid valve replacement.

**Figure 9:**
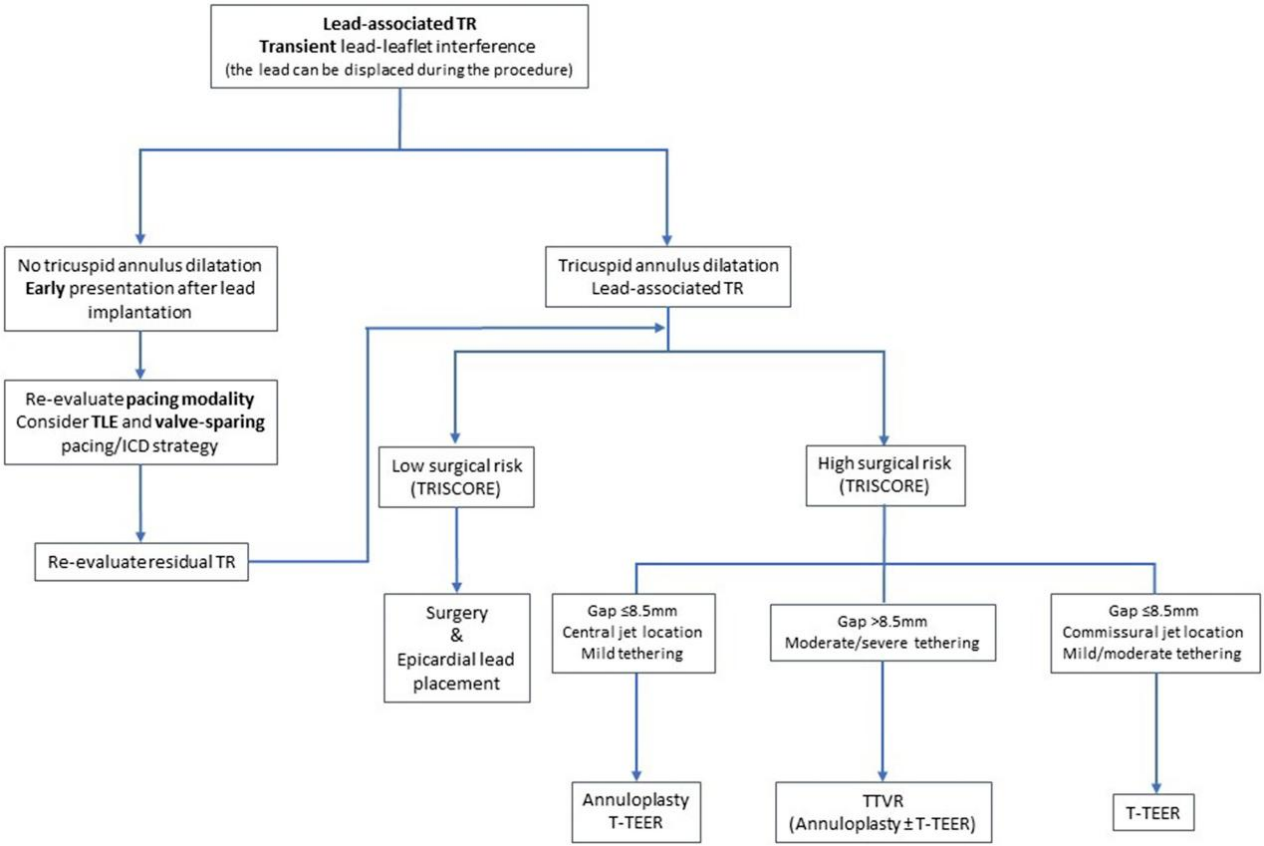
Treatment algorithm of cardiac implantable electronic device-associated tricuspid regurgitation. ICD, implantable cardioverter defibrillator; TEER, transcatheter edge-to-edge repair; TLE: transvenous lead extraction; TR, tricuspid regurgitation; TTVR, transcatheter tricuspid valve replacement.

A treatment algorithm of CIED-related and CIED-associated TR is proposed in *Figures 8* and *9*. Multidisciplinary counselling in an extended Heart Team including electrophysiologists with specific expertise in device and TLE, interventional cardiologists specialized in the treatment of the atrioventricular valves, and cardiac surgeons is of paramount importance. Futility should be excluded in elderly patients at advanced stage.

Although not able to revert or avoid disease progression, medical treatment, mainly diuretics, is useful to improve right heart failure symptoms. However, according to the current guidelines in the absence of advanced RV dysfunction or severe pulmonary hypertension (especially precapillary or unmitigable PH), none of these therapies should delay referral for surgery or transcatheter intervention.^74^

#### Transvenous lead extraction

It may seem very compelling to treat lead-related TR with TLE. Since there are no specific recommendations on whether or not to perform TLE in patients with relevant TR, it is important to undertake a thorough risk–benefit analysis. This interdisciplinary analysis performed by a specific Heart Team should evaluate the case-based probability of improving or worsening TR and weigh the inherent risks of the procedure against potential complications associated with lead jailing. As a prerequisite for this analysis, TR mechanism has to be carefully evaluated using echocardiography to provide evidence of a lead-related TR cause, as opposed to simple association due to annular dilatation.^49^

Polewczyk *et al*.^75^ studied the effects of TLE procedures in 119 patients with lead-related TV dysfunction in an overall cohort of 2678 patients. They reported TR improvement in only a minority of patients (35%) with lead-related TR after TLE, which was associated with better long-term survival. Nazmul *et al*.^76^ published the results of a case series on TLE procedures to improve symptomatic CIED-associated TR with limited success rates and no improvement in 75% of cases. This emphasizes the importance of a precise assessment of the cause of TR and rapid intervention, if causality is established by imaging. In patients with pre-existing tricuspid annular dilatation, isolated TLE of a transvalvular lead is unlikely to result in TR improvement. While no specific cut-offs have been defined for these indications so far, the ones mentioned in the current guidelines regarding concomitant tricuspid annuloplasty (≥40 mm or >21 mm/m^2^ using 2D echo) may provide useful guidance.^74^

Although rather infrequent, severe injuries to the TV apparatus can occur during TLE and an incidence of 2.5% has been reported among more than 2600 procedures.^77^ Another study observed acute TR change, defined as a ≥1-grade increase, in 11.5% of the 208 examined patients.^78^ In both studies, a longer lead dwell time was a risk factor for TLE-related acute TR worsening. There are insufficient data to indicate if specific TLE techniques (i.e. mechanical sheaths, laser sheaths, and femoral snares) increase the risk of valve dysfunction.

The role of TLE as a preparation for transcatheter therapies to avoid lead jailing remains unclear. The decision to use TLE should rely on an individual, case-based Heart Team evaluation. In a different scenario, the 2017 Heart Rhythm Society expert consensus on CIED lead management gives a Class I recommendation based on expert opinion for lead extraction in patients with planned stent deployment in a vein to avoid entrapment of the lead.^79^

#### Surgical management

Historically, isolated TV surgery has been associated with high rate of early mortality (8%–10% in most series).^80,81^ However, according to recent evidence with a strong focus on pre-operative patient optimization and selection, isolated TV surgery has achieved far better results, in particular when patients are operated early. A recent international multicentre study on 426 patients reported a 30-day mortality 5.8% in an ‘all-comers, all-aetiologies, and all-stage’ population: repair techniques,^82^ beating heart strategy,^83^ and non-endocarditis etiology^84^ were described in different analyses as prognostic modifiers for long-term survival. Data on the surgical management of lead-related TR are scarce. Pfannmueller^15^ reported acceptable freedom from TR recurrence at 5 years after surgical tricuspid repair in the presence of a PPM lead. However, in this series, a limited number of patients suffered from lead-related TR, and in this subgroup, the authors strongly suggest lead removal followed by epicardial repositioning or implantation into the coronary sinus. The same group reported a 6.4% 30-day mortality and 58% survival at 5 years of isolated TV surgery in patients with pacemaker leads.^85^ Encouraging data come from Saran *et al*.,^86^ who reported favourable outcomes of CIED-related TR (*n* = 349) vs. CIED-associated TR (*n* = 249) with a 30-day mortality of 4.4% vs. 9.5% (*P* < .05) and increased late survival despite a higher replacement rate in the lead-related TR group.

Irrespective of TR aetiology, patient selection, RV optimization, risk estimation using dedicated scores (TRI-SCORE^87^ or LaPar^88^), and early treatment represent the key factors to improve outcomes of isolated TV surgery.^89^

The presence of transvalvular leads poses a technical challenge in patients undergoing surgical TV repair because leads may restrict, perforate or adhere to valve leaflets,^60^ or inadvertently dislocate during the operation. If the transvalvular lead does not interfere with leaflet function (lead-associated TR), an isolated TV annuloplasty may suffice to achieve a durable result and similar survival at 5 years compared with patients undergoing repair in the absence of a lead.^90^ In contrast, if the lead is adherent to or perforates a leaflet, separation from the leaflet by blunt or sharp dissection is needed with possible subsequent damage to the leaflet tissue requiring direct reconstruction using autologous or bovine pericardial patches.^86^ An undersizing annuloplasty is almost always part of the procedure.^76,91^

When lead interference and leaflet damage are detected, repositioning of the lead into the posteroseptal or the anteroposterior commissure should be considered.^86,92^ Lead wrapping with annular and/or leaflet material before either ring or prosthesis implantation may even allow for future lead removal without creating relevant paravalvular leakage.

However, complex pathologies may require early replacement to avoid additional clamp time. If a pacemaker is required after TV replacement, alternative valve-sparing strategies should be chosen, in particular coronary sinus pacing,^73^ while transprosthetic lead placement should be the exception but may be associated with acceptable results.^93^

#### Transcatheter edge-to-edge repair

Like for any other repair technique, the evaluation for tricuspid transcatheter edge-to-edge repair (TEER) must take into account the potential role of the lead in the TR mechanism. The role of 3D echocardiography is of utmost importance to identify the underlying mechanisms. Two main scenarios can be identified:

- The lead is an innocent bystander without a causative role for TR (CIED-associated) and is not localized in the grasping zone or immediate catheter trajectory: in this case, TEER can be performed using a traditional approach, according to the valve anatomy, regurgitation location, and coaptation gap. This is typically the case for leads localized in the posteroseptal commissure.
- The lead is mobile but represents the main cause of TR (CIED-related) or contributes to it: in this case, lead mobilization prior to or during the procedure can be attempted using the TEER system itself or alternatively an additional steerable sheath. Lead immobilization either in one of the commissures or between two clips can occur and is generally without further consequences. Lead extraction before the intervention can also be considered, particularly in cases where PPM implantation is recent.

Although no relevant differences regarding procedural results and short clinical outcomes after TEER were reported between patients with or without leads,^94^ potential lead–clip interactions need to be taken into consideration. Depending on the amount of slack, the relationship to the lead may change in an upright position. Despite the frequent close proximity of clips to implanted leads, no relevant lead damages or malfunctions have been reported after TEER.

#### Direct transcatheter annuloplasty

Direct transcatheter annuloplasty showed favourable outcomes and sustained TR reduction in patients with severe symptomatic TR at high risk for TV surgery.^95^ No adverse effects of pre-existing CIED leads on procedural and clinical success were demonstrated.^96^ However, several lead-associated aspects should be considered: navigation of the implant system in the right atrium and around the tricuspid annulus is challenging. Knowledge of the exact lead position in relation to guide catheter and valve apparatus is of high importance to prevent periprocedural interaction. Computed tomography and 3D echocardiography should be critically assessed before the intervention to define position and mobility of leads as well as potential impingement of leaflets. If a lead is causally related to TR due to impingement of leaflets in the absence of pronounced annular dilation, the indication for annuloplasty as first-line therapy should be critically questioned.

To avoid adverse interaction with the implant system and enable complete navigation options, CIED leads must be crossed anteriorly within the right atrium before steering towards the annulus.

#### Orthotopic tricuspid valve replacement

First-in-human experience with the EVOQUE system (Edwards Lifesciences, USA) showed procedural success in all patients with PPM leads (36% of the study population) with no or mild residual TR and no procedure-related or device-associated PPM dysfunction.^97^ Two patients (8%) developed conduction disturbances requiring permanent PPM implantation. The larger TRISCEND I study confirmed the efficacy of valve replacement with the EVOQUE valve in 176 patients of whom 32.4% had a CIED lead. The most common complications were severe bleeding (25.5% at 1 year) and PPM implantation (13.3% at 30 days). Other devices with less radial forces, like the LuX valve (Jenscare, China), may have lower PPM rates.^98^

#### Heterotopic bicaval valve implantation

This technique, currently considered as palliative option in patients with advanced disease, has recently shown to increase quality of life and functional capacity.^99^ In the TRICUS EURO study, 23% of the patients treated with the TricValve system (Products & Features, Germany) had PPM implanted prior to the index procedure, and no CIED-related adverse events were observed. The implantation of caval valve systems has been shown to be feasible in patients with one or several leads but results in extensive lead entrapment in the superior vena cava, which is discouraged by the 2017 Heart Rhythm Society expert consensus statement.^79^

#### Considerations around lead jailing during tricuspid valve interventions

**Figure 10:**
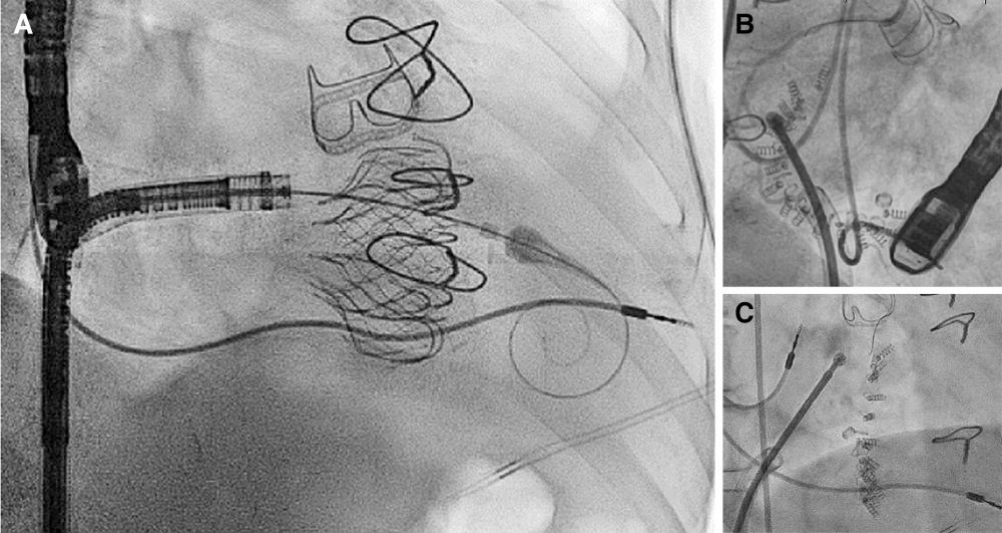
Examples of lead jailing during trancatheter tricuspid valve interventions. Examples of pacemaker lead jailing during transcatheter valve replacement with the EVOQUE system (*A*) and direct transcatheter tricuspid annuloplasty with the Cardioband system (*B* and *C*). In both cases, no lead dysfunction was detected up to 2 years after the procedure.

Examples of lead jailing during transcatheter TV interventions are shown in *Figure 10*. Several considerations are required to assess the feasibility of lead jailing during pre-procedural planning.

Device interrogation is an essential step to guide decision-making, ensure the safety of the procedure, and detect potential immediate and longer-term changes after lead jailing. The ventricular pacing dependency, battery status, lead impedance, and atrial and ventricular pacing thresholds should be systematically assessed. The arrhythmia burden should also be carefully reviewed to anticipate the risk associated with a potential ICD dysfunction after lead jailing.

### Management of conduction disorder following tricuspid valve interventions

#### Risk of lead dysfunction

**Figure 11:**
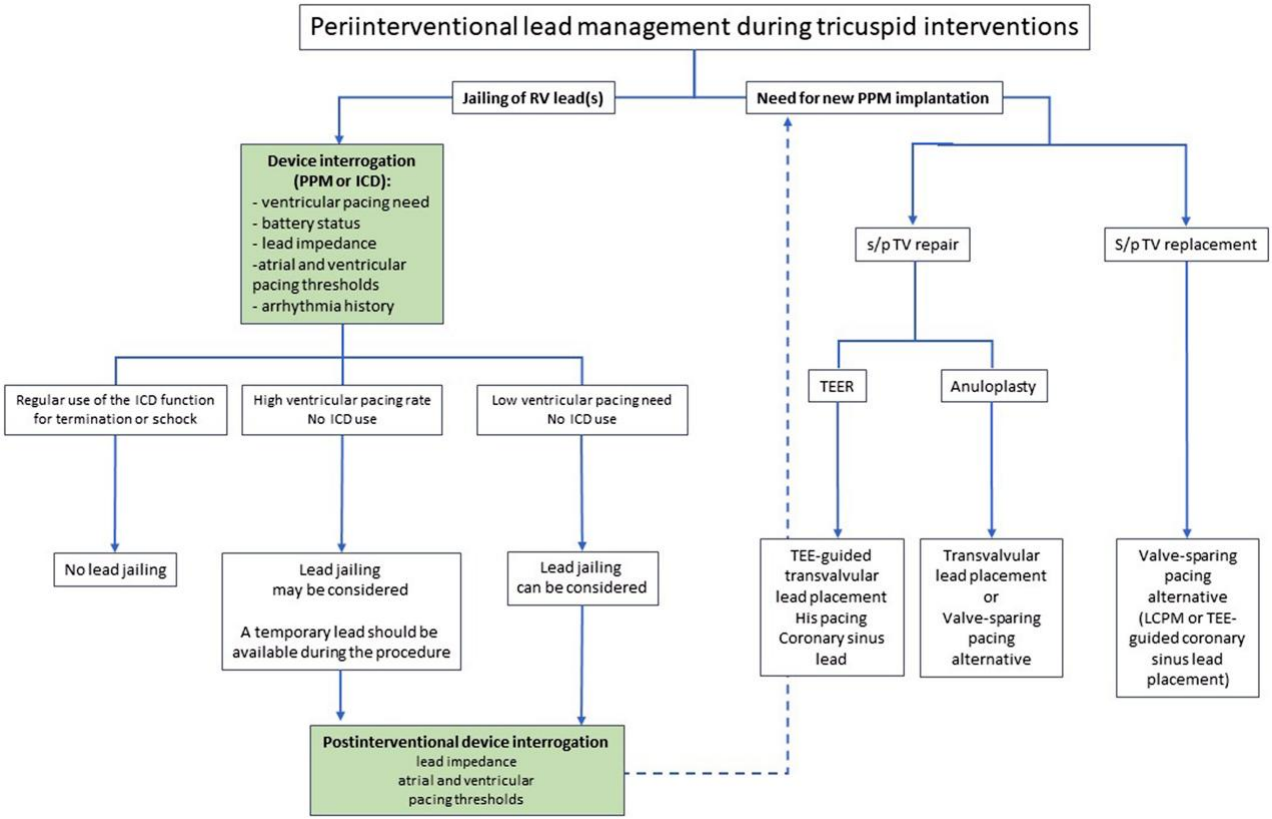
Peri-interventional lead management during tricuspid interventions. ICD, implantable cardioverter defibrillator; LCPM, leadless cardiac pacemaker; PPM, permanent pacemaker; RV, right ventricle; s/p, status post; TEE, transoesophageal echocardiography; TEER, transcatheter edge-to-edge repair; TV, tricuspid valve.

There are very limited data on the impact of TV interventions in patients with an existing PPM or ICD lead. Apart from case reports and small series without lead dysfunction,^93,100,101^ the Valve-in-Valve International Database reviewed 329 patients with prior TV repair or replacement who subsequently underwent valve-in-valve or valve-in-ring transcatheter TV replacement.^102^ The cohort included 31 (9%) patients with an existing transvenous lead that crossed the TV. In three patients, the lead was extracted prior to TTVR; in the other 28 patients, the RV lead was jailed between the new valve and the degenerated one. During a mean follow-up of 15.2 months, three patients (11%) suffered a lead-related complication (acute lead dislodgement; marked increase in pacing impedance and pacing threshold 2 weeks after TTVR; and lead fracture 7 months after TTVR). Fractures from ICD lead have also been described after transcatheter valve replacement, and, while the pacing function can be easily substituted, restoration of the defibrillator function may require complex alternative options as described above. Therefore, jailing of an ICD used for arrhythmia termination or shock therapy should not be recommended (*Figure 11*).

#### Risk of cardiac implantable electronic device infection

Cardiac implantable electronic device infections have a reported prevalence of 1%–3% within a year of implantation^103^ and are associated with increased mortality.^104^ Risk factors for CIED infection include renal dysfunction, diabetes, younger age, the presence of more than two leads, and heart dysfunction. Whether the infection rate is impacted by lead jailing is unknown.

The infection of a system with one or several jailed leads that cannot be extracted represents a particularly delicate situation.^105^ Therefore, a careful interdisciplinary risk–benefit analysis is needed when lead jailing is anticipated taking into account the fact that elderly patients undergoing transcatheter tricuspid procedures have usually a rather long lead dwell time increasing the risk of TLE. Any infective endocarditis complication arising after the procedure will imply surgical material removal, in particular after valve replacement, which may not be appropriate with regard to the excessive surgical risk and will need to be managed conservatively in many cases.

#### Risk of new conduction disturbances during and after tricuspid valve interventions

**Figure 12:**
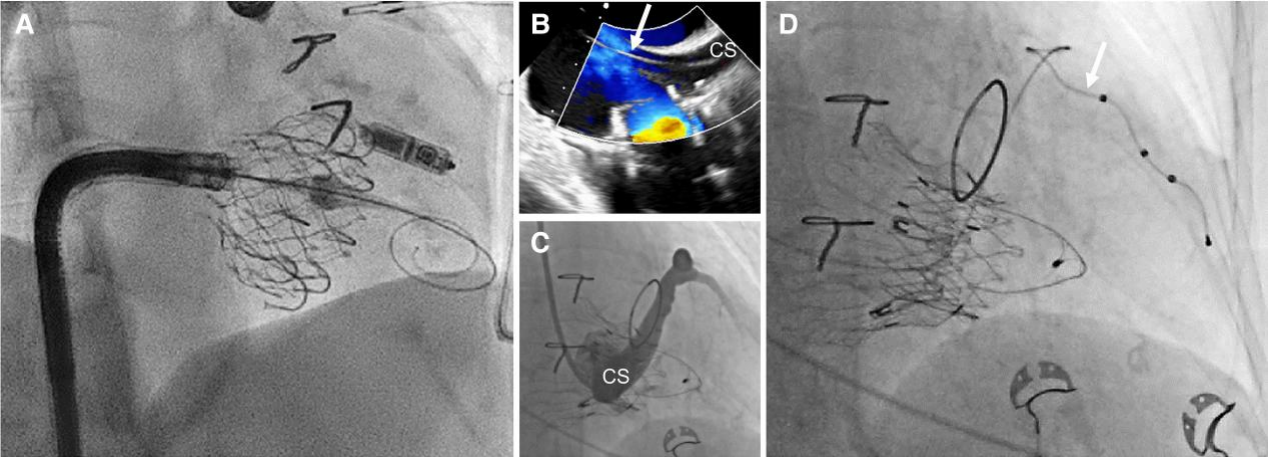
Examples of valve-sparing pacing strategies in the context of transcatheter tricuspid valve replacement. (*A*) Pre-emptive implantation of a LCPM before transcatheter tricuspid valve replacement with the EVOQUE system in a patient with a complete atrioventricular block during right heart catheterization. The position of the LCPM needs to be carefully chosen; (*B*) transoesophageal echocardiography guiding of coronary sinus lead implantation (white arrow) after transcatheter tricuspid valve replacement with the LUX valve; (*C*) sinus angiography highlighting potential interaction between the bioprosthesis and the coronary sinus; and (*D*) final pacemaker lead (white arrow) placement into the coronary sinus. CS, coronary sinus.

Given the target region of TV interventions, atrioventricular conduction disturbances may be expected when replacement is used, probably depending on the amount of radial force and oversizing used for valve anchoring. Surgical data do indeed suggest repair may be less vulnerable to atrioventricular block as compared with replacement, with a 9% vs. 21% pacemaker implantation event rate (odds ratio 0.37, 95% confidence interval 0.24–0.58) in a meta-analysis of >15 000 procedures.^106^ Very little systematic data are available given the fact transcatheter tricuspid therapy remains in its early stages, although cases of complete atrioventricular block have been described with edge-to-edge repair and Cardioband. When a pacemaker indication becomes evident, alternative valve-sparing strategies (*Table 3* and *Figure 11*) or epicardial lead placement should be considered over conventional RV lead position to avoid downstream reintroduction of lead-related complications (*Figure 12*), even if transvalvular lead implantation after tricuspid annular percutaneous annuloplasty with the Cardioband system and edge-to-edge repair is less problematic. Patients requiring pacemaker implantation after TV replacement should be evaluated for LCPM or lead implantation into the coronary sinus that has been shown safe and reliable in patients with TV disease.^107^ The threshold for using TEE guiding for device implantation should be low. Alternatively, His-bundle pacing may also avoid crossing the tricuspid annulus although experience with both approaches is limited.^108^

### Summary and recommendations (quality of evidence in brackets B = observational data; C = expert consensus)

#### Imaging and cardiac implantable electronic device implantation

- Echocardiography should be performed before and after CIED implantation; patients at risk should follow up on a regular basis (C).
- Specific implantation techniques to avoid valve interaction or damage are recommended (prolapsing technique) (C).
- Novel CIEDs may improve outcome due to ‘valve sparing’ approaches (His-bundle pacing, LCPMs, and coronary sinus lead implantation) (B).

#### Work-up of cardiac implantable electronic device patients with severe tricuspid regurgitation

- Patients with severe tricuspid disease and CIED leads in place should be referred to expert centres for workup and therapy (C).
- 3D echocardiography and cardiac CT are required to understand the interaction of CIED leads and the TV (C).
- Device interrogation to understand its use is essential (C).
- It is of utmost importance to evaluate the RV function and pulmonary artery pressure to estimate the risk of surgery/intervention (B).

#### Treatment of severe tricuspid valve disease in patients with cardiac implantable electronic device

- Risk assessment with dedicated risk scores (e.g. TRI-SCORE) is required (B).
- Transvenous lead extraction should be considered early since TR improvement late after implantation and in patient with annular dilatation is unlikely (B).
- Depending on risk and lead characteristics, TLE can be considered to facilitate tricuspid procedures (C).
- Low surgical risk patients should undergo surgery at an expert centre with specific post-operative care protecting the right ventricle (C).
- Novel transcatheter therapies are increasingly applied in these mostly older and multimorbid patient population with promising results (C).

## Conclusion

In conclusion, patients with CIEDs are at increased risk to develop TV disease and need regular echocardiographic follow-ups. If significant TV disease develops, early referral to an expert center and an interdisciplinary approach is mandatory to initiate timely treatment. Surgery and the emerging transcatheter therapies work in synergy to prevent irreversible damage of the heart and other organs and may improve patient’s quality of life and prognosis.

## Supplementary data

Supplementary data are available at *European Heart Journal* online.

## Supplementary Material

ehad783_Supplementary_Data

## Data Availability

No data were generated or analysed for this manuscript.

## Pre-registered Clinical Trial Number

None supplied.
