# Correction to: Tricuspid valve disease and cardiac implantable electronic devices

**DOI:** 10.1093/eurheartj/ehae221

**Published:** 2024-03-29

This is a correction to: Martin Andreas *et al.*, Tricuspid valve disease and cardiac implantable electronic devices, *European Heart Journal*, Volume 45, Issue 5, 1 February 2024, Pages 346–365, https://doi.org/10.1093/eurheartj/ehad783

In the originally published version, the nineteenth author was incorrectly listed as Piotr Rudzinski. The article has been updated to list the author as Piotr Nikodem Rudzinski.

