# Supplementary material for: Tricuspid valve disease and cardiac implantable electronic devices
Source: Eur Heart J. 2023 Dec 14;45(5):346–65. doi: 10.1093/eurheartj/ehad783 (PMC10834167; doi:10.1093/eurheartj/ehad783)
Supplement: ehad783_Supplementary_Data [file ehad783_supplementary_data.zip › ehad783_Supplementary_Data.docx]

**Supplementary material**

**Supplementary section 1: Methodology**

This review article was developed with electrophysiologists, interventional cardiologists, imaging specialists, and cardiac surgeons during a structured discussion process. Multidisciplinary expert groups defined by the PCR Tricuspid Focus Group met with electrophysiologists and other dedicated experts during several plenary online sessions and smaller expert groups meetings to collect and discuss relevant evidence, reach consensus and draft the manuscript that aims to formulate clinical and scientific conclusions. Recommendations on patient management and treatment are proposed.

**Supplementary section 2: Leadless pacemakers and CRT**

LCPMs have been introduced since two decades and provide VVI, VDD (with hemodynamic sensing of atrial contraction) or DDD pacing (with wireless communication between right atrial and right ventricular units)(1-4). The use of the dual chamber leadless pacemaker (Aveir, Abbott Medical) has been recently shown to be safe and to provide at least 70% atrioventricular synchrony in 97% of the patients(4).

The WISE-CRT system is a leadless unit with an electrode implanted endocardially into the left ventricle and activated through an ultrasound signal triggered by a standard CRT system(5) or alternatively a LCPM(6).

**Supplementary section 3: Subcutaneous ICDs**

Boston Scientific (Marlborough, MA) subcutaneous ICD (S-ICD) system includes a generator placed in the left lateral thorax between the latissimus dorsi and serratus anterior muscles, connected to a lead tunnelled in the subcutaneous pre-sternal tissue. The device has received CE mark in 2009 and has since evolved to achieve comparable safety and efficacy compared to standard transvenous devices. Although only able to deliver shock therapy, wireless coupling with a LCPM enables bradycardia pacing and the ability to deliver antitachycardia pacing (ATP) via LCPM is being currently assessed. However, there are a number of shortcomings which still need to be addressed, such as improvement in diagnostics and enhanced programming options for troubleshooting rhythm discrimination(7). More recently, the Medtronic (Minnesota, MA) extra-vascular ICD (EV-ICD) has been introduced and combines a subcutaneous generator placed in the left lateral thorax, connected to a lead tunnelled beneath the sternum. This device not only provides shock therapy, but also ATP due to its proximity to the heart. Although initial results are encouraging, capture thresholds are elevated, pacing may be painful (resulting rarely in inactivation of ATP), and inappropriate shock rates are high (close to 10%) due to issues with atrial and noise oversensing(8). Future generations of the system are likely to address these limitations.

**Supplementary section 4: Innovative alternative strategies for ICD therapy**

DF-1 ICDs dissociate low- and high-voltage components of the lead and offer more options than DF-4 systems which combine both entities(9).

1) Standalone ICD coil implantation in the azygos vein or coronary sinus (connected to the RV port), and coupled with antero-lateral subcutaneous “SQ” array (connected to the superior vena caval port) to enhance the shock vector. With this approach, ventricular sensing and pacing is ensured by an epicardial pacing lead tunneled to the generator, or by a coronary sinus pacing lead(10).

2) Placement of a standard ICD lead in the middle cardiac vein(11). Ventricular sensing and pacing may be performed by this lead, but adequate electrical parameters (with lack of diaphragmatic myopotential oversensing or phrenic capture) should be verified, or alternatively provided by a coronary sinus lead connected to the RV port of a DF-1 ICD.

3) ICD lead screwed in the low right atrium, with ventricular sensing and pacing provided by a coronary sinus or epicardial lead(12).

4) Implantation of SQ arrays or ICD lead/coils (with epicardial pacing leads) directly on the ventricular surface. However, long-term results are missing.

Defibrillation testing should be performed in all cases with non-conventional ICD configurations.

**References**

1. Reddy VY, Exner DV, Cantillon DJ, Doshi R, Bunch TJ, Tomassoni GF, et al. Percutaneous Implantation of an Entirely Intracardiac Leadless Pacemaker. N Engl J Med. 2015;373(12):1125-35.

2. Reynolds DW, Ritter P. A Leadless Intracardiac Transcatheter Pacing System. N Engl J Med. 2016;374(26):2604-5.

3. Boersma LV, El-Chami M, Steinwender C, Lambiase P, Murgatroyd F, Mela T, et al. Practical considerations, indications, and future perspectives for leadless and extravascular cardiac implantable electronic devices: a position paper by EHRA/HRS/LAHRS/APHRS. Europace. 2022;24(10):1691-708.

4. Knops RE, Reddy VY, Ip JE, Doshi R, Exner DV, Defaye P, et al. A Dual-Chamber Leadless Pacemaker. N Engl J Med. 2023.

5. Auricchio A, Delnoy PP, Butter C, Brachmann J, Van Erven L, Spitzer S, et al. Feasibility, safety, and short-term outcome of leadless ultrasound-based endocardial left ventricular resynchronization in heart failure patients: results of the wireless stimulation endocardially for CRT (WiSE-CRT) study. Europace. 2014;16(5):681-8.

6. Carabelli A, Jabeur M, Jacon P, Rinaldi CA, Leclercq C, Rovaris G, et al. European experience with a first totally leadless cardiac resynchronization therapy pacemaker system. EP Europace. 2020;23(5):740-7.

7. Friedli A, Burri H. S-ICDs: advantages and opportunities for improvement. Expert Rev Med Devices. 2022;19(3):237-45.

8. Friedman P, Murgatroyd F, Boersma LVA, Manlucu J, O'Donnell D, Knight BP, et al. Efficacy and Safety of an Extravascular Implantable Cardioverter-Defibrillator. N Engl J Med. 2022;387(14):1292-302.

9. Burri H, Starck C, Auricchio A, Biffi M, Burri M, D'Avila A, et al. EHRA expert consensus statement and practical guide on optimal implantation technique for conventional pacemakers and implantable cardioverter-defibrillators: endorsed by the Heart Rhythm Society (HRS), the Asia Pacific Heart Rhythm Society (APHRS), and the Latin-American Heart Rhythm Society (LAHRS). Europace. 2021;23(7):983-1008.

10. Blank E, Shah AD, Rosenblum JM, Lloyd MS. "Valve-sparing" transvenous defibrillator systems after tricuspid valve intervention. Heart Rhythm. 2021;18(12):2212-4.

11. Lopez JA. Implantable cardioverter defibrillator lead placement in the middle cardiac vein after tricuspid valve surgery. Europace. 2012;14(6):853-8.

12. Biffi M, Bertini M, Ziacchi M, Boriani G. Transvenous cardioverter-defibrillator implantation in a patient with tricuspid mechanical prosthesis. Journal of cardiovascular electrophysiology. 2007;18(3):329-31.
